# Effect of adeno-associated virus (AAV)-mediated overexpression of PEPCK-M (Pck2) on Clenbuterol-induced muscle growth

**DOI:** 10.1371/journal.pone.0218970

**Published:** 2019-06-25

**Authors:** David M. Loczenski-Brown, Sarah Jones, Jeni Luckett, Zoe Daniel, Madelaine C. Brearley, Francis J. P. Ebling, Tim Parr, John M. Brameld

**Affiliations:** 1 School of Biosciences, University of Nottingham, Sutton Bonington Campus, Loughborough, United Kingdom; 2 School of Medicine, University of Nottingham Medical School, Nottingham, United Kingdom; 3 School of Life Sciences, University of Nottingham Medical School, Nottingham, United Kingdom; West Virginia University, UNITED STATES

## Abstract

We previously identified PEPCK-M (encoded by the Pck2 gene) to be highly up-regulated in skeletal muscle of pigs treated with Ractopamine, an anabolic beta-adrenergic receptor agonist. To determine whether PEPCK-M had a causative role in modulating the skeletal muscle growth response to Ractopamine, we used adeno-associated virus 1 (AAV1) to over-express Pck2 (AAV-Pck2) in murine skeletal muscle. A contralateral limb design was employed, such that each mouse served as its own control (injected with a GFP-only expressing AAV1, labelled AAV-GFP). Daily injections of Clenbuterol (1 mg/kg for 21 days) or vehicle control were also carried out to assess the effects of AAV-Pck2 overexpression on the anabolic response to a beta-adrenergic agonist. AAV-Pck2 overexpression in leg muscles of male C57BL6/J mice for 4 weeks (6–10 weeks of age) increased Pck2 mRNA (~100-fold), protein (not quantifiable) and enzyme activity (~3-fold). There was a trend (p = 0.0798) for AAV-Pck2 overexpression to reduce TA muscle weights, but there was no significant effect on muscle fibre diameters or myosin heavy chain isoform (MyHC) mRNA expression. When skeletal muscle growth was induced by daily administration of Clenbuterol (for 21 days), overexpression of AAV-Pck2 had no effect on the growth response, nor did it alter the expression of Phosphoserine Aminotransferase-1 (Psat1) or Asparagine Synthetase (Asns) mRNA or the Clenbuterol-induced decreases in MyHC IIa and IIx mRNA expression (p = 0.0065 and p = 0.0267 respectively). However AAV-Pck2 overexpression reduced TA muscle weights (p = 0.0434), particularly in the Control (vehicle treated) mice (p = 0.059 for AAV x Clenbuterol interaction) and increased the expression of Seryl-tRNA Synthetase (Sars) mRNA (p = 0.0477). Hence, contrary to the original hypothesis, AAV-Pck2 overexpression reduced TA muscle weights and did not mimic or alter the muscle hypertrophic effects of the beta-adrenergic agonist, Clenbuterol.

## Introduction

Skeletal muscle constitutes a large component of total body mass and is a major contributor to the regulation of systemic metabolism and locomotive capabilities. A decline in skeletal muscle mass, such as that encountered with ageing, disuse or chronic disease [1] has been attributed to reduced quality of life and in some cases is considered detrimental to disease prognosis. Whilst exercise and nutrition are known beneficial regulators of musculoskeletal health, pharmacological interventions to improve skeletal muscle mass remain an unmet clinical need. Furthermore, the use of pharmacological agents in the livestock industry to improve the efficiency of lean deposition has received renewed interest in light of a potential food security crisis by 2050, as published in the Foresight Report [2,3].

Previously, we used a transcriptomic screening approach to identify novel genes associated with beta-adrenergic agonist-induced muscle growth in pigs. We showed that Pck2 (which encodes Phosphoenolpyruvate Carboxykinase 2; PEPCK-M) was strongly up-regulated, at both the mRNA and protein levels, in skeletal muscle of pigs treated with Ractopamine, a beta-adrenergic agonist [4]. PEPCK catalyses the conversion of oxaloacetate to phosphoenolpyruvate, which can be used for gluconeogenesis, synthesis of anabolic intermediates (such as serine and glycine), and recycling of TCA cycle anions for energy generation [5]. There are two PEPCK enzymes that are encoded by distinct genes, Pck1 and Pck2. The cytosolic form, PEPCK-C (Pck1), has been heavily investigated for its role in gluconeogenesis in the liver [5]. Although not a gluconeogenic tissue, there is low but significant expression of PEPCK-C in muscle [6]. When this isoform was specifically over expressed in skeletal muscle by generating transgenic mice, they had an enhanced exercise capacity relative to controls and their muscles had higher mitochondrial numbers as well as an elevated triglyceride content [6]. This indicated that there was a re-patterning of energy metabolism toward a more oxidative metabolism, with increased use of fats as substrate. However, the mitochondrial form, PEPCK-M (Pck2), has received little attention until recently. A number of studies now report a role for PEPCK-M (Pck2) in proliferative cell growth [7–9] and it has become an attractive metabolic drug target in relation to several cancers. Over-expression of Pck2 in dividing cells increases proliferation, whereas knockdown or inhibition of Pck2/PEPCK-M reduces cell proliferation [7–9]. Pck2 is lowly expressed in adult skeletal muscle [10], possibly because it is a terminally differentiated tissue, so the increased expression of Pck2 mRNA and protein following administration of a beta-adrenergic agonist to pigs was particularly profound. The role of Pck2 in a terminally differentiated tissue, such as skeletal muscle, remains unclear.

To determine whether Pck2/PEPCK-M has a causative role in modulating skeletal muscle growth, we established an *in vivo* model using adeno-associated virus 1 (AAV1) to over-express Pck2 in murine skeletal muscle. We then determined whether AAV-Pck2 over-expression in skeletal muscle would influence beta-adrenergic (clenbuterol)-induced muscle growth in mice.

## Materials and methods

### Animals

**Fig 1:**
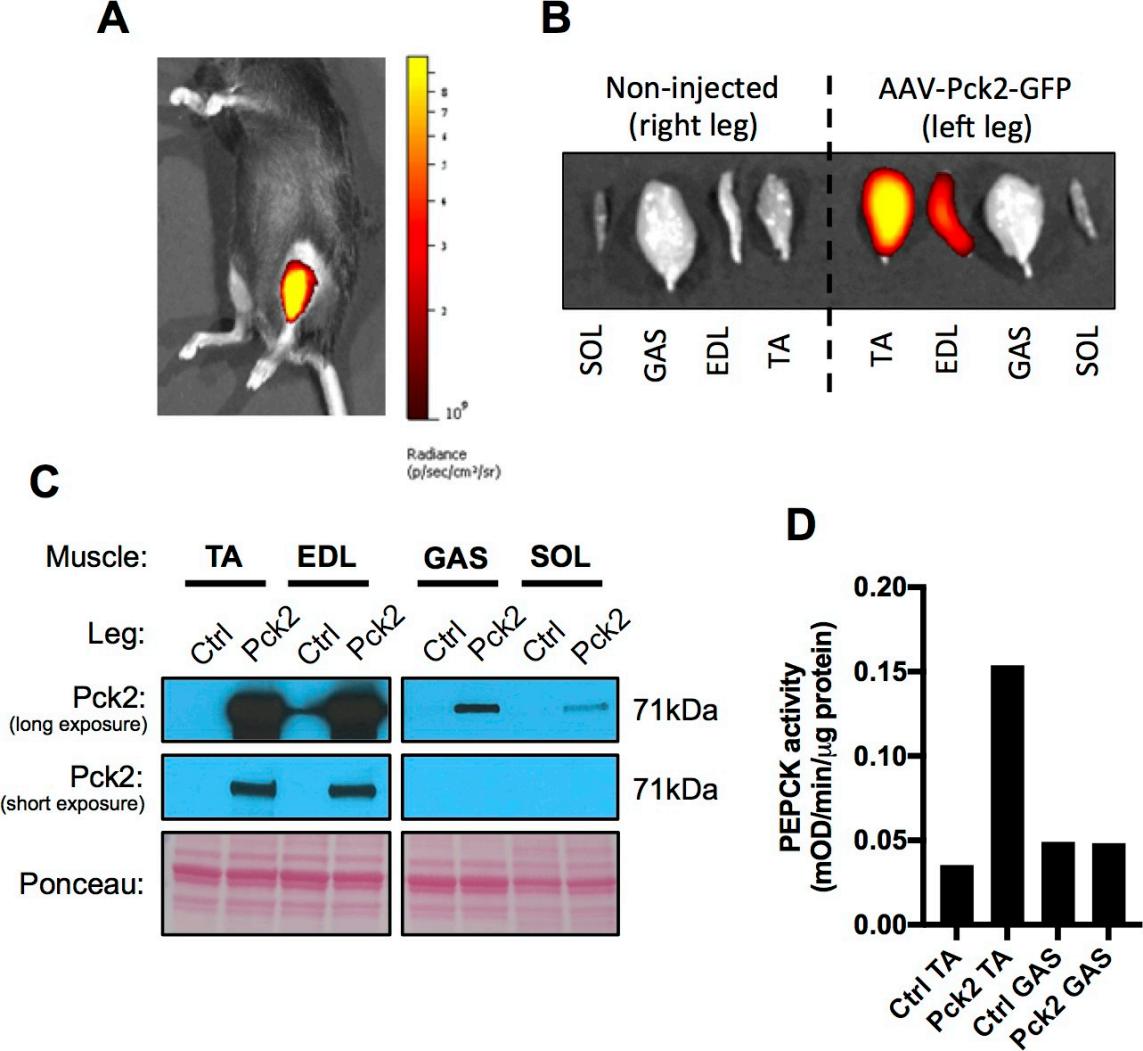
Validation of AAV contralateral limb study design (pilot; *n* = 1). (A) GFP signal 3 weeks post injection of AAV-Pck2-GFP into the TA muscle. (B) GFP signal of dissected lower limb muscles from the left (AAV-Pck2-GFP injected) and right (non-injected) legs. (C) PEPCK-M protein expression in lower limb muscles from the left (AAV-Pck2-GFP injected) and right (non-injected) legs. (D) PEPCK enzyme activity in the Tibialis anterior (TA) and Gastrocnemius (GAS) muscles from the left (AAV-Pck2-GFP injected) and right (non-injected) legs.

**Fig 2:**
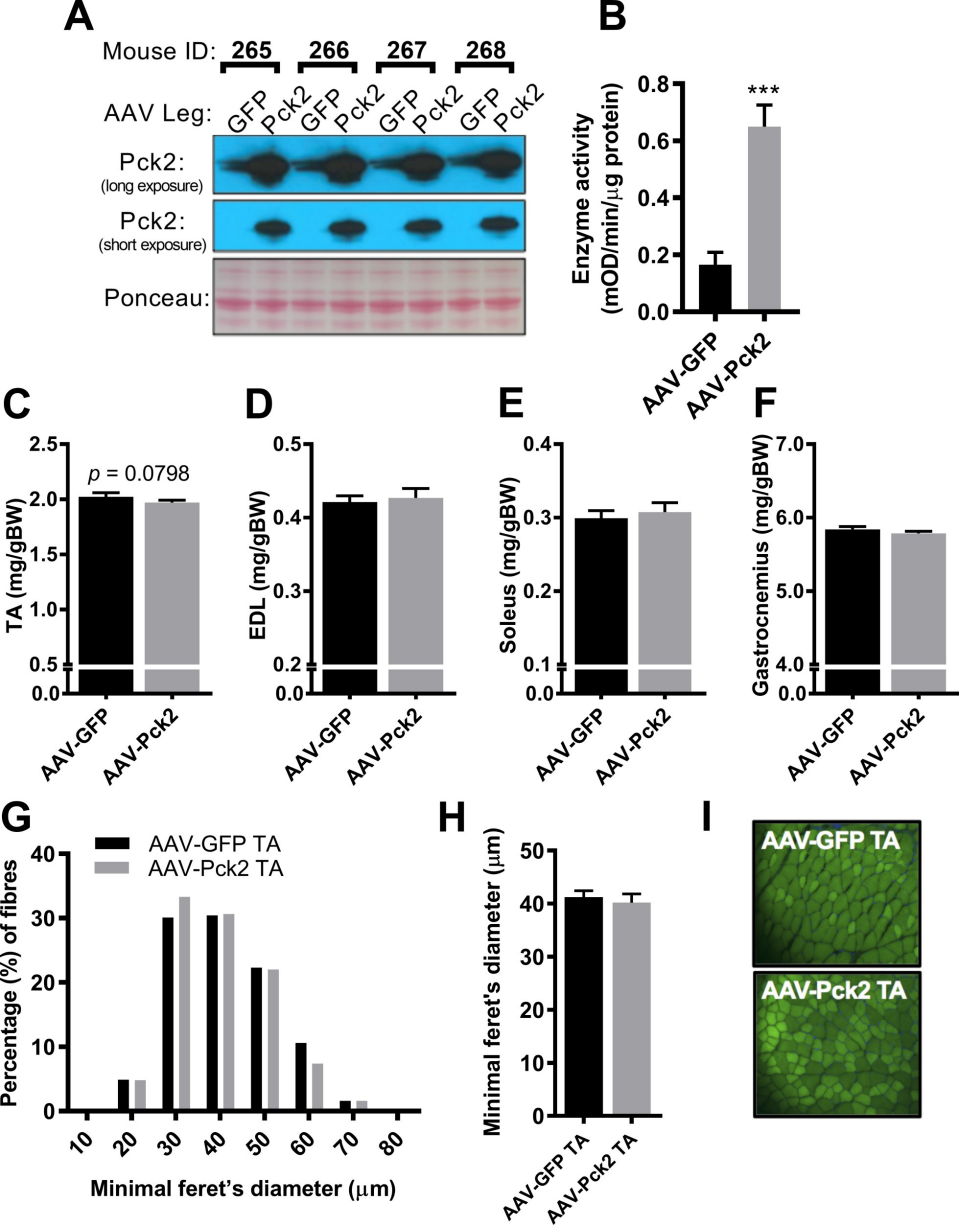
Effect of AAV-mediated over-expression of Pck2 on the TA muscle 4 weeks post injection. (A) Effect of AAV-Pck2 on PEPCK-M protein expression (n = 4). (B) PEPCK enzyme activity in the tibialis anterior muscle 4-weeks following AAV-Pck2 injection (n = 4). Effect of AAV-Pck2 on the weights of the (C) tibialis anterior (TA), (D) Extensor digitorum longus (EDL), (E) Soleus and (F) Gastrocnemius muscles (n = 8, except n = 7 for EDL). Effects of AAV-Pck2 on (G) % distribution and (H) average minimal feret’s diameter of muscle fibres from the tibialis anterior muscle (n = 4). (I) Representative images of cross-sections from AAV-infected tibialis anterior muscles. *** p<0.001 indicates a significant effect of AAV-Pck2 compared to AAV-GFP.

**Fig 3:**
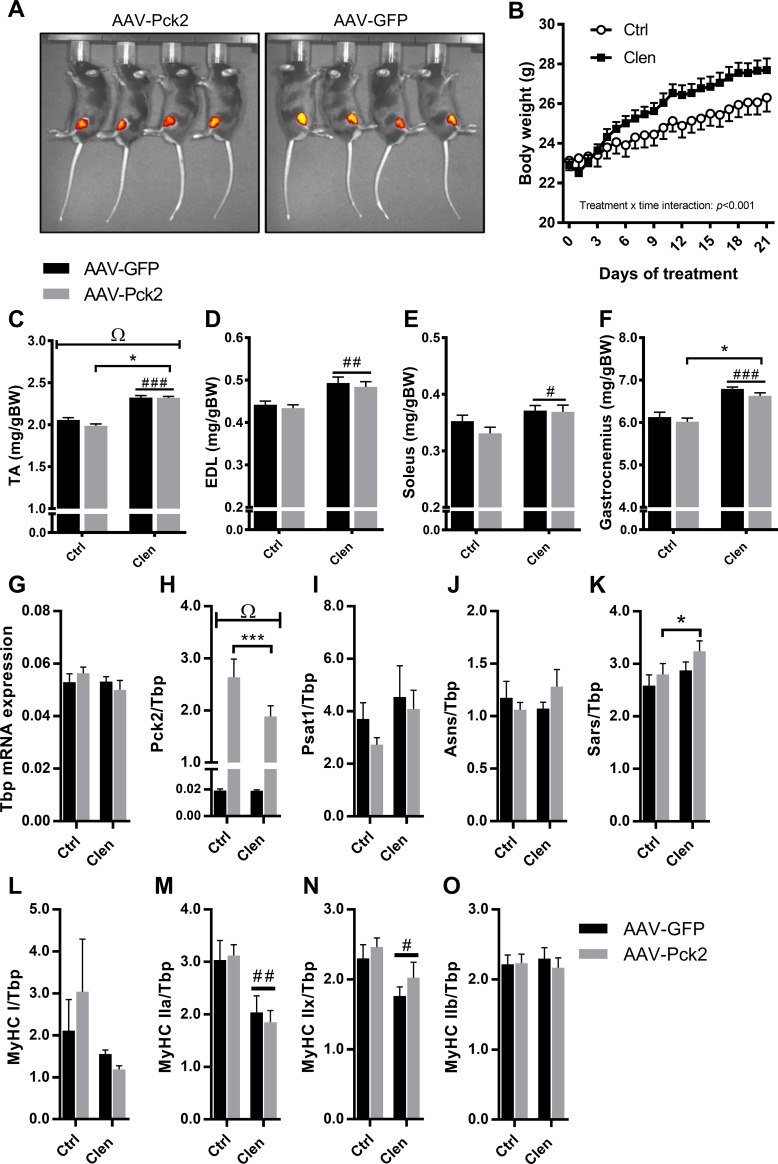
Effect of AAV-mediated Pck2 over-expression on Clenbuterol-induced muscle growth and gene xpression. (A) Representative images of GFP signal from the left and right legs of AAV-GFP and AAV-Pck2-GFP infected mice 7 days after intramuscular injection. (B) Effect of Clenbuterol (Clen) or phosphate buffered saline (Ctrl) on daily body weight starting 7 days post AAV injections (n = 8). (C-F) Effect of AAV-Pck2 on Clenbuterol mediated increases in muscle weights (Tibialis anterior, TA; Extensor digitorum longus, EDL; Soleus; Gastrocnemius; n = 8 except n = 7 for EDL Clen group). (G) TATA-box binding protein (Tbp) mRNA expression, showing no effects of AAV-Pck2 or Clenbuterol treatment. Effects of AAV-Pck2 and Clenbuterol on (H) Pck2, (I) Phosphoserine Aminotransferase-1 (Psat1), (J) Asparagine Synthetase (Asns), (K) Seryl-tRNA Synthetase (Sars) and myosin heavy chain (MyHC) types I (L), IIa (M), IIx (N) and IIb (O) mRNA expression in TA muscle. All data (except Tbp mRNA) was normalized to the reference gene, Tbp (n = 8 for all). Ω p<0.09 indicates a trend for an AAV x treatment interaction. * p<0.05 and **** p<0.001 indicate a significant effect of AAV-Pck2 compared to AAV-GFP. # p<0.05, ## p<0.01 and #### p<0.001 indicate a significant effect of Clen compared to Ctrl treated mice.

Male C57BL6/J mice (aged 5–6 weeks; Charles River UK Ltd.) were used for all experiments. Mice were group housed (*n* = 4 per cage) under a controlled temperature (21 ± 1°C) and held on 12h light: 12h dark photoperiod, lights off at 19:00, with *ad libitum* access to rodent chow (Teklad 2018, Envigo, UK) and filtered water. All mice were ear notched for identification purposes. The pilot experiment (Fig 1) was conducted using 1 mouse. Effects of AAV-Pck2 on muscle mass (Fig 2) was assessed on 8 mice. Effects of AAV-Pck2 on Clenbuterol-mediated muscle growth (Fig 3) was conducted on 16 mice (*n* = 8 per treatment group). Treatment groups for the Clenbuterol experiment were mixed within and between cages and mice were allocated to treatment groups such that starting mean body weight was not statistically different between groups.

All animal procedures were approved by The University of Nottingham Ethical Review Committee and conducted in accordance with the UK Animals (Scientific Procedures) Act of 1986 (Project License PPL PFBB3B51F).

### Adeno-associated virus (AAV)

pAAV-CMV-Pck2-T2A-emGFP (abbreviated to AAV-Pck2) and pAAV-CMV-emGFP (abbreviated to AAV-GFP) were produced and packaged into adeno-associated virus 1 (AAV1) by Cyagen Biosciences, using the *mus musculus* phosphoenolpyruvate carboxykinase 2 (mitochondrial) (Pck2) mRNA sequence (Accession number NM_028994.2). The viral T2A peptide sequence between Pck2 and emGFP was used to allow for co-translational cleavage of both proteins at near equimolar levels and hence GFP signal could be used as a surrogate marker of Pck2 expression [11]. AAV’s were stored in 150mM NaCl, 2mM MgCl_2_, 50mM Tris (pH 8.0) at -80°C for less than 1 year and thawed on ice on the day of use.

### Intramuscular AAV injections

Whilst under recoverable anaesthesia (using 1.5% Isofluorane), mice were administered a single intramuscular injection of 20μl containing ~1.8 x 10^10^ viral genomes (rAAV1) into the left and right *Tibalis Anterior* (TA) muscles. The syringe needle (29G x 13mm) was inserted parallel to the muscle fibre orientation directly into the mid-belly of the TA muscle. AAV-Pck2-GFP was injected into the left TA whereas AAV-GFP was injected into the right TA to serve as a contralateral control. Following the injection, mice remained anaesthetized for ~3 minutes to prevent the AAV leaking from the injection sites when the muscles become loaded. Mice were then recovered from anaesthesia and maintained in their home cages for 3 weeks (pilot experiment) or 4 weeks. There were no adverse effects of the injection procedure.

### IVIS Spectrum optical imaging and Clenbuterol injections

To test the effect of AAV-Pck2 during accelerated muscle growth, over-expression of Pck2 was first confirmed prior to administration of the beta-2 adrenergic agonist, Clenbuterol. Seven days following the AAV injections, mice were imaged under recoverable anaesthesia (1.5% isoflurane) on an IVIS Spectrum imaging system (PerkinElmer). GFP signal was used as an indicator of successful delivery to the TA muscles and AAV function (i.e. translation of AAV-delivered genes). Following confirmation of localized GFP expression, mice were injected daily with Clenbuterol (CAS# 21898-19-1; Sigma Aldrich UK) at 1 mg/kg via the subcutaneous route for 3 weeks.

### Termination

Mice were euthanized by cervical dislocation. The Tibialis anterior (TA), Extensor digitorum longus (EDL), Gastrocnemius and Soleus muscles were dissected and weighed. The proximal end (~20mg) of the TA muscle was carefully dissected (transverse cut) using a sharp scalpel blade and used for protein, mRNA or enzyme activity analysis, whilst the remaining distal end of the TA was embedded in OCT and frozen in dry-ice cooled isopentane for the analysis of muscle fibre diameters.

### Muscle fibre diameter analysis

Sections from the mid-belly of the TA muscle were cryosectioned at -24°C, at a thickness of 12μm. Sections were mounted with Fluoroshield overnight. Five fields of view (each containing ~100 fibres) from a single section per muscle were imaged at 20x magnification. Sections from 4 TA muscles per treatment were analysed, which equated to ~2000 fibres per treatment being measured. Muscle fibre perimeters were manually traced (using AAV-derived GFP signal to define the muscle fibres) and the minimal feret’s diameter was calculated using image-J software. Minimal feret’s diameter of individual muscle fibres is considered superior to measuring muscle fibre cross-sectional area because it is less influenced by differences in the orientation of the muscle when sectioning [12]. The treatment groups were blinded to the investigator during the analysis.

### Protein expression by western blotting

Protein was extracted from the Tibialis anterior (TA) muscle by homogenization in extraction buffer containing 150mM NaCl, 50mM HEPES, 2.5mM EDTA, 10% Glycerol, 1% Triton, and protease and phosphatase inhibitor cocktails (Roche, UK). The homogenate was centrifuged at ~15,000g for 10 minutes and the supernatent transferred to an equal volume of 2x Laemmli loading buffer. Constant protein was loaded and separated on a 4–15% precast acrylamide gel (Criterion TGX, BioRad) and wet transferred to nitrocellulose membranes, with ponceau staining used to confirm the equal loading of protein. Membranes were then probed with an anti-PEPCK-M antibody (Cell Signaling #6924), followed by an anti-rabbit HRP-conjugated secondary antibody (GE Healthcare Life Science). Bands were visualized using ECL detection reagent (GE Healthcare Life Science) and exposure to photographic film in a dark room. Densitometry of band intensity was conducted to determine protein expression levels.

### PEPCK enzyme activity

Frozen TA muscle was powdered in a liquid nitrogen cooled pestle and mortar, followed by homogenisation (using a rubber pestle and mortar followed by brief sonication) in 3 volumes of extraction buffer (10mM HEPES, 1mM EDTA, 1mM DTT, protease and phosphatase inhibitors). Debris was pelleted by centrifugation for 15 minutes at 14,000 rpm at 4°C and the supernatant retained. Protein quantification was determined by the Lowry assay [13]. Protein extracts were freeze-thawed once prior to assay to facilitate lysis of the mitochondria. Approximately 20μg protein was used per reaction, with activity normalised for actual protein content after the assay. PEPCK activity was assessed in the direction of oxaloacetate formation, as described elsewhere [14], with some modifications. Reactions were performed in duplicate in a volume of 200μl (using a 96-well clear assay plate) containing 100mM Imidazole Cl (pH 6.8), 3mM MnCl2, 10mM Phenylalanine, 30mM NaHCO3, 0.15mM NADH, 2mM PEP, 5.46μg/ml MDH, 3mM MgSO4, and 37.3mM HEPES. This buffer was gassed with CO_2_ for 10 minutes prior to the addition of Deoxy-GDP (final concentration 0.5mM). The drop in absorbance (i.e. NADH) was measured every 30 seconds for 20 minutes (i.e. 40 reads) to determine the total velocity in mOD/min.

### Gene expression analysis

Total RNA was extracted from 20mg of powdered TA muscle using the RNeasy Fibrous Tissue Mini Kit (Qiagen) according to manufacturer’s instructions. First strand cDNA was synthesised from 500ng RNA using the Transcriptor First Strand cDNA synthesis Kit (Roche) according to manufacturer’s instructions. Quantitative-RT-PCR was conducted using the SYBR-green method as previously described [15]. Relative transcript abundance was determined from a standard curve and corrected against TATA-box binding protein (Tbp) mRNA expression (which was unaffected by treatment). Adult myosin heavy chain (MyHC) mRNA isoforms were used as an indicator of changes to muscle fibre type composition [14]. Oligonucleotide sequences for MyHC isoforms [15] and Pck2 [16] have been published previously.

Tbp oligonucleotide sequences were F: 5’-AGAATAAGAGAGCCACGGACAACT-3’ and R: 5’-GCTAGTCTGGATTGTTCTTCACTCTTG-3’.

### Implementation of the NC3R’s (www.nc3rs.org.uk/the-3rs)

1. To reduce animal numbers by 50%, each mouse served as its own control by injecting the contralateral limb with a control AAV expressing emGFP only.
2. Non-invasive confirmation of GFP expression (as a marker of AAV-derived Pck2 expression) ensured that mice only began receiving Clenbuterol if the AAV infection was successful.

### Statistical analyses

Statistical analyses were performed in Prism (Version 7). The effects of AAV-Pck2 were assessed between the experimental (left) leg and the contralateral control (right) leg by a paired t-test. Effects of AAV-Pck2 and Clenbuterol were assessed by two-way analysis of variance (ANOVA), blocking for animal (to account for the legs being from the same animal). The effect of Clenbuterol on daily body weight was analysed by two-way repeated measures ANOVA. Bonferroni or Sidak’s post hoc analysis was conducted where appropriate. Statistical significance was accepted if *p*<0.05. All data are presented as mean ± standard error of the mean (SEM).

## Results

A pilot study showed successful transduction of the TA and EDL muscles 3 weeks following injection with AAV-Pck2 (Fig 1; n = 1). The GFP signal from the peripherally located TA muscle was detectable through shaved skin using the IVIS Spectrum optical imager (Fig 1A). Post mortem analysis of dissected muscles showed that the GFP signal was largely restricted to the TA and EDL muscles of the injected leg only (Fig 1B), with no GFP detected in the non-injected leg muscles. Over-expression of the PEPCK-M protein (encoded by Pck2) was successfully elevated in muscles of the injected leg only (i.e. not in the control non-injected leg), with over-expression predominantly observed in the TA and EDL muscles and limited over-expression in the Gastrocnemius (GAS) and Soleus (SOL) muscles (Fig 1C). Elevated PEPCK-M protein expression in the TA muscle coincided with a 3-fold increase in PEPCK enzyme activity (Fig 1D), demonstrating the functional efficacy of AAV-Pck2 to increase PEPCK activity in muscle. Collectively, these pilot results show that viral particles injected into the left TA muscle disseminate locally (i.e. to adjacent muscles only), but are restricted to the injected leg.

Based on the pilot study, we then carried out a contralateral control limb study and showed that AAV-Pck2 increased PEPCK-M protein levels compared to the contralateral limb injected with AAV-GFP (Fig 2A; n = 4), which corresponded to a 3-fold increase in PEPCK activity in the TA muscle (Fig 2B; p<0.001; n = 4). AAV-Pck2 caused a small but not quite statistically significant reduction in TA muscle weights (Fig 2C; p = 0.0798), whereas it had no effect on other lower limb muscle weights (Fig 2D–2F). Muscle fibre diameters of the TA were not altered by AAV-Pck2 administration (Fig 2G and 2H), although there was a slight decrease in the percentage of larger (60μm) fibres and a slight increase in smaller (30μm) fibres (Fig 2G). Representative images (Fig 2I) of TA muscle cross sections injected with AAV-Pck2 or AAV-GFP demonstrate the high transduction efficiency of AAV1 to infect nearly 100% of muscle fibres.

To test the effects of AAV-Pck2 overexpression during accelerated muscle growth, Clenbuterol administration was initiated 7 days following AAV-Pck2 and AAV-GFP injections, when a localised GFP signal was detected in the TA muscle (Fig 3A). GFP signal from the TA muscle was used as a marker of AAV delivered Pck2/GFP gene expression, as described in the methods section (Fig 3A). Daily Clenbuterol administration then commenced from day 7 onwards for 3 weeks (days 0–21 in Fig 3B), which caused a progressive increase in body weight with time (Fig 3B; treatment x time interaction p<0.001). AAV-Pck2 caused a significant reduction in TA muscle weights (Fig 3C; p = 0.0434), particularly in Control (vehicle treated) mice (p = 0.059 for AAV x Clenbuterol interaction). As expected, Clenbuterol increased TA (Fig 3C; p<0.001), EDL (Fig 3D; p<0.01), Soleus (Fig 3E; p<0.05) and Gastrocnemius (Fig 3F; p<0.001) muscle weights, but there were no effects of AAV-Pck2 on the EDL, Gastrocnemius or Soleus muscle weights (Fig 3C–3F; p>0.05).

There were no significant effects of AAV-Pck2 or Clenbuterol nor any interaction on TATA-box binding protein mRNA (Fig 3G), which was subsequently used as the reference gene for normalisation. Pck2 mRNA expression was increased 100-fold by AAV-Pck2 overexpression (Fig 3H; p<0.001), but Pck2 mRNA was slightly reduced by Clenbuterol administration compared to Control mice (Fig 3H; p = 0.085 for treatment and p = 0.084 for interaction). There were no effects of AAV-Pck2 overexpression or Clenbuterol treatment on either Phosphoserine Aminotransferase-1 (Psat1) or Asparagine Synthetase (Asns) mRNA (Fig 3I and 3J). In contrast, AAV-Pck2 overexpression significantly increased Seryl-tRNA Synthetase (Sars) mRNA (Fig 3K; p = 0.0477), but there was no effect of Clenbuterol, nor any interaction. Clenbuterol significantly reduced mRNA expression of myosin heavy chain IIa (MyHC IIa; Fig 3M; p<0.01) and MyHC IIx (Fig 3N; p<0.05), but not the other adult myosin heavy chain (MyHC) isoforms (Fig 3L and 3O). AAV-Pck2 overexpression did not alter mRNA expression of any of the MyHC isoforms (Fig 3L–3O) and there were no interactions.

## Discussion

Using AAV1 we were able to overexpress Pck2 mRNA and PEPCK-M protein and thereby increase PEPCK enzyme activity in selected skeletal muscles of mice, in the locality of the injection site. Overexpression of PEPCK-M (Pck2) in male C57BL6/J mice for 4 weeks (6–10 weeks of age) tended or significantly reduced TA muscle weights, but appeared to have little effect on muscle fibre diameters. Like PEPCK-C, PEPCK-M transcripts and protein are only expressed at low levels in adult muscles [10]. Transgenic mice overexpressing PEPCK-C specifically in skeletal muscle had a very distinctive hyperactive phenotype, associated with an increased capacity to utilise fatty acids for respiration, particularly at exercise intensities that would be expected to require carbohydrate [6]. In the current study, Pck2/ PEPCK-M overexpression was localised to only a few leg muscles, so was very unlikely to influence whole-body respiration, hence this was not assessed. In PEPCK-C transgenic mice, neither muscle fibre diameter nor myosin heavy chain expression were determined, but there was a visible increase in triglyceride content in muscles, which was not observed in this study in muscles overexpressing of PEPCK-M/ Pck2. In addition, muscles from the muscle specific PEPCK-C transgenic mice had increased mitochondrial density and high succinate dehydrogenase enzyme activities [6], which suggests that their muscle fibres had become oxidative type fibres. Expression of myosin heavy chain isoform mRNA normally reflects the metabolic characteristics of the muscle, and thereby their fibre type [17]. The lack of any changes in myosin heavy chain isoform expression in the current study suggests that AAV-Pck2 overexpression did not alter muscle fibre type, at least not in the timeframe investigated.

We have previously described the effects of beta-adrenergic agonists on various species, including pigs [4], sheep [18] and mice [16]. These agents increase muscle weights with an associated increase in fast MyHC isoform expression and decrease in slow MyHC, reflecting a characteristic shift to a faster fibre type [4, 18]. In addition, we have recently shown that beta-adrenergic agonist stimulated muscle growth in pigs is associated with increased expression of genes associated with serine/one-carbon/glycine (SOG) biosynthesis [4], amino acid biosynthesis, protein translational capacity and an integrated stress response [19]. It has been postulated that PEPCK-M is an enzyme capable of redirecting TCA cycle intermediates into the SOG biosynthesis pathway [9]. In Ractopamine treated pigs, both PEPCK-M and the first enzyme in the SOG pathway, PHGDH, were co-ordinately up regulated [4]. The current study clearly shows that overexpression of Pck2 by itself reduced muscle growth (in mice), contrary to our original hypothesis. We also postulated that AAV-Pck2 overexpression might prime muscle metabolism so that there would be an accentuated growth response to Clenbuterol. Skeletal muscle growth was induced by daily administration of Clenbuterol, but AAV-Pck2 overexpression did not enhance the muscle growth or gene expression response to Clenbuterol. Instead, AAV-Pck2 overexpression tended to reduce muscle weights, particularly in the absence of Clenbuterol. The increased expression of Pck2/ PEPCK-M previously reported in skeletal muscle of pigs administered Ractopamine [4] is therefore unlikely to play a causative role in muscle growth. It is possible that a more chronic over-expression of AAV-Pck2 (i.e. beyond 4 weeks) might have caused a differential phenotype to those reported herein, however, from our experience, 4 weeks is sufficient to detect muscle anabolic or catabolic effects of pharmacological agents in mice [16]. It is also likely that the biology underpinning skeletal muscle growth in pigs and mice differs mechanistically, as we have observed clear differences in the response to beta-adrenergic agonists in the two species. In pigs we observed significant changes in the expression of genes associated with an integrated stress response [4, 19], whilst we saw no induction of the ISR gene program in mice [16]. It is possible that mice are already metabolically stressed (due to their high metabolic rate), so respond to beta adrenergic agonists without inducing the ISR gene program. Indeed our unpublished data indicates that the serine biosynthesis pathway genes are more highly expressed in mouse compared to pig muscles. Hence pigs might potentially respond differently to AAV-Pck2 over-expression than observed here for mice.

## Conclusion

Contrary to our original hypothesis (based on previous pig studies), AAV-Pck2 overexpression in skeletal muscle of mice appeared to reduce muscle growth and had little effect on the response to the beta-adrenergic agonist, Clenbuterol. However the effect of a beta-adrenergic agonist on skeletal muscle gene expression, particularly Pck2 mRNA, appears to differ between mice and pigs, suggesting that pigs might potentially respond differently to AAV-Pck2 over-expression.
